# Evaluation of fecal samples as a valid source of DNA by comparing paired blood and fecal samples from American bison (*Bison bison*)

**DOI:** 10.1186/s12863-019-0722-3

**Published:** 2019-02-26

**Authors:** David Forgacs, Rick L. Wallen, Amy L. Boedeker, James N. Derr

**Affiliations:** 1 Interdisciplinary Graduate Program of Genetics, Texas A&M University, College Station, TX 77843 USA; 2 Department of Veterinary Pathobiology, Texas A&M University, College Station, TX 77843 USA; 3 National Park Service, Yellowstone National Park, Hot Springs, Mammoth, WY 82190 USA

**Keywords:** Bison, Yellowstone National Park, Fecal DNA, Microsatellite, STR, Heterozygosity, Allelic dropout

## Abstract

**Background:**

The collection and analysis of fecal DNA is a common practice, especially when dealing with wildlife species that are difficult to track or capture. While fecal DNA is known to be lower quality than traditional sources of DNA, such as blood or other tissues, few investigations have verified fecal samples as a valid source of DNA by directly comparing the results to high quality DNA samples from the same individuals. Our goal was to compare DNA from fecal and blood samples from the same 50 American plains bison (*Bison bison*) from Yellowstone National Park, analyze 35 short tandem repeat (STR) loci for genotyping efficiency, and compare heterozygosity estimates.

**Results:**

We discovered that some of the fecal-derived genotypes obtained were significantly different from the blood-derived genotypes from the same bison. We also found that fecal-derived DNA samples often underestimated heterozygosity values, in some cases by over 20%.

**Conclusions:**

These findings highlight a potential shortcoming inherent in previous wildlife studies that relied solely on a multi-tube approach, using exclusively low quality fecal DNA samples with no quality control to account for false alleles and allelic dropout. Herein, we present a rigorous marker selection protocol that is applicable for a wide range of species and report a set of 15 STR markers for use in future bison studies that yielded consistent results from both fecal and blood-derived DNA.

**Electronic supplementary material:**

The online version of this article (10.1186/s12863-019-0722-3) contains supplementary material, which is available to authorized users.

## Background

Genetic variation is the basis for evaluating biodiversity within and between populations; without genetic variation, populations could not evolve or adapt to changing environmental conditions [1]. Modern wildlife conservation is becoming increasingly more dependent on the use of genetic tools due to their efficiency and reliability in identifying species and relationships within and between populations, and even resolving taxonomic conflicts. Collecting fecal material as a non-invasive sampling strategy has been widely used in situations where capturing wildlife is dangerous, time consuming, or unfeasible [2, 3]. Some researchers have warned against using fecal DNA without first conducting a pilot study to assess genotyping error rates [4, 5]; most studies, however, adapt or develop markers for use with fecal DNA without validating the markers by using paired DNA from a high quality source (e.g. blood, soft tissue). Instead, they opt for a multi-tube approach that involves repeat testing of the same fecal samples to estimate genotyping errors rather than direct comparison of low versus high quality DNA isolated from the same individuals [6–10], with few exceptions [11–15]. Thus, without a direct validation of the fecal-derived alleles, uncertainty arises about how allelic dropout and false allele rates could bias the reported results. Some studies compared results from genotyping fecal samples under different conditions [16–18] and different methods for DNA extraction, such as rehydrating and carefully removing only the mucosal coat of the scat [19] or using a magnetic bead protocol [20], but to our knowledge, a direct and comprehensive comparison between a large number of paired samples at numerous loci has not been performed in any wildlife species.

The two most common issues with using low quality samples identified by previous STR studies are allelic dropout and false allele rates [21]. Allelic dropout occurs when heterozygotes are mistakenly reported as homozygotes due to the stochastic loss of one of the two alleles during amplification. Allelic dropout can lead to underestimating heterozygosity and genetic diversity, as well as misjudging the level and direction of natural selection [5, 21]. In wildlife studies using fecal DNA markers, allelic dropout was often high, determined as 8% in a study on mountain lions [11], 11.1% in wolves [22], and up to 49% in chimpanzees [23]. However, all of these studies inferred dropout rates by re-amplifying DNA from the same fecal samples several times and comparing those results (the multi-tube approach); none of them used matching high quality DNA samples for comparison.

False allele rate (misprinting) describes a scenario where neither of the two alleles present in an individual amplifies, and instead, completely different alleles are detected. Misprinting can be due to PCR error, DNA degradation, or contamination. False alleles can also bias population metrics by providing inaccurate genetic diversity, heterozygosity, and population structure estimates, as well as overestimating census size because different samples from the same animal can appear to be unique. False allele rates in previous studies have ranged from negligible in coyotes [13] and dolphins [14] to 5.6% in wolves [22], and 18.3% in barbary macaques [24].

The use of short tandem repeats (STRs), commonly known as microsatellites, is a well-established approach to analyzing non-coding repetitive regions for genetic analyses [25, 26]. STRs are typically considered to be neutral markers that are not generally subject to strong selection [27], therefore, they are especially useful for population genetic and forensic analyses due to the high variability in the number of repeats between individuals [28]. Thus, by using just a handful of STR loci, low probability of identity (P_ID_) and low likelihood of odds (LOD) scores can be achieved [29]. For population-level analyses, that means the probability of two animals possessing the exact same genotype across all markers by mere chance is low.

In order to assess the efficiency of STR markers with fecal DNA, we analyzed paired fecal and blood samples from American plains bison (*Bison bison bison*) from Yellowstone National Park. Yellowstone bison have been at the forefront of numerous conflicts for the past 150 years with regards to population management and genetic diversity [30–33]. In order to better understand the complete range of genetic diversity in Yellowstone bison, it is necessary to establish a truly random sampling practice. Previous approaches to estimate diversity indices have been heavily reliant on opportunistic sampling: when a portion of the population moves beyond the park boundary, bison are subject to capture and removal, thus animals that are sampled are ultimately the ones eliminated from the population [34–36]. While these studies provide valuable preliminary data on the genetic status of Yellowstone bison, the use of non-invasive methods would provide a unique opportunity to sample a larger and more representative proportion of the population. Previous analysis using fecal DNA from Yellowstone bison has been conducted, but the lack of validation using paired high quality DNA samples and the low number of loci tested necessitated further research [37]. If a set of validated markers was established for use with bison fecal DNA, then the opportunity to further explore the genetic diversity of the Yellowstone population and reevaluate information that was previously collected could be used to improve our understanding of this unique bison population. It could also prove useful with other free-ranging bison herds, such as the ones at Elk Island National Park and Wood Buffalo National Park in Canada, and the Henry Mountains bison herd in Utah.

The objective of our research was to verify fecal samples as a reliable source of DNA by comparing 50 fecal samples from Yellowstone bison with blood samples from the same animals. We looked at the rates of allelic dropout and false alleles, as well as the overall feasibility of using fecal matter as the source of DNA for future genetic analyses in this population. In addition, most wildlife research currently lacks information regarding the validity of the STR markers used with fecal samples due to a lack of comparison to high quality DNA samples from the same animals. Here, we present a case study to show the importance and necessity of the meticulous design and validation of markers.

## Methods

Fifty bison from the Yellowstone National Park bison herd that ventured outside the park were captured during January and February of 2015. The 41 females and 9 males ranged 1–4 years in age. Fecal samples were obtained directly from the rectum and stored at − 20 °C in 95% ethanol. Simultaneously, blood was drawn from the same fifty animals and spotted on Whatman FTA (Flinders Technology Associates) cards (GE Health Care, USA) (Pairs 1–50, see Additional file 1). Fecal DNA extractions were performed with the QIAamp Fast DNA Stool Kit (Qiagen, Valencia, California, USA), following the manufacturer’s protocols. Blood samples were extracted from the FTA cards by soaking the individual 1.20 mm punches in 200 μL of 20 mM NaOH followed by incubation at 50 °C for 30 min with periodic inversion. After aspirating the NaOH, 200 μL of 10% TE buffer was added to the punches and then removed after 2 min at room temperature. The punches were left uncovered to dry overnight and were used directly in the PCR reaction.

Both fecal and blood samples went through the same PCR protocol, adapted from Schnabel, 2001 [38] and Halbert, 2003 [35]. The 35 STR markers that were used are listed in Additional file 2. PCR was run with 3.175 μL of fluorescently labeled stock primer mix (concentrations listed in Additional file 2), 0.5 μL of 10X MasterAmp (Epicentre Technologies, Madison, Wisconsin, USA), 0.25 μL of 10 mM dNTPs, and 0.075 μL of Promega GoTaq (Promega, Madison, Wisconsin, USA) which was added to 1 μL of fecal DNA extract or FTA punch. During PCR, the samples were heated for 3 min at 96 °C, followed by 4 cycles of 20 s at 96 °C, 30 s at 58 °C, and 90 s at 65 °C, decreasing the initial temperatures by 1 °C per cycle. Then the samples were subjected to 26 cycles of 20 s at 96 °C, 30 s at 54 °C, and 90 s at 65 °C. The reaction was concluded with 1 min at 96 °C, 1 min at 54 °C, and 20 min at 65 °C. For all fecal samples, a second PCR was set up with the same parameters, using 1 μL of amplified DNA from the previous PCR reaction as the template for the second round in order to obtain the sufficient amount of copies for genotyping.

After the amplification of the STR markers, the samples were genotyped using the AB Genetic Analyzer 3130xl. The results were analyzed using GeneMapper v. 3.7 software (Applied Biosystems, Carlsbad, CA) and the genotyping calls were made separately for fecal and blood samples to avoid any bias. When the fecal/blood pairs from the same animals were compared for each STR locus, they were noted as matching (same genotype for both), not matching (different genotype) or unreadable (either blood or fecal sample has yielded no intelligible data). All mismatches were verified using the multi-tube approach by a minimum of two independent amplification and genotyping runs. In the case of the Y chromosome marker INRA189, even a single instance of amplification of the allele was scored as a male.

A final STR panel was chosen based on the ability to genotype fecal DNA with the highest reliability (> 95% matches) (Table 1). These loci were mapped to the *Bos taurus* (UMD 3.1) reference genome and the genetic distance was determined based on the 1.23–1.25 cM/Mb recombination frequency reported in domestic cattle [39, 40]. The expected frequency of each allele was determined based on 10,000–17,000 plains bison from across North America previously genotyped at each locus (Derr, unpublished). The probability of identity (P_ID_) was calculated by assuming the most conservative estimate that a bison was homozygous for the most common allele at each locus, following the directions in Butler, 2005 [41] (Additional file 3). All the linked loci were less than 50 map units apart, so only the loci with the lowest major allele frequencies were included in the probability of identity calculation from each chromosome, providing the most conservative estimate.

**Table 1:** List of 15 STR loci chosen due to their high fidelity and efficiency

Marker	Percent matching (excluding reruns)	Percent matching (including reruns)	Chromosome	Major allele frequency (x_i_)	Major genotypic frequency (x_i_^2^)
BM7145	100%	100%	1	0.800	0.640
BM4307	100%	100%	1	0.782	0.612
BM2113	96%	96%	2	0.360	0.129
CSSM42	100%	100%	2	0.607	0.368
AGLA293	100%	98%	5	0.958	0.917
SPS113	98%	98%	10	0.613	0.376
BL1036	100%	100%	14	0.313	0.098
BM4513	100%	98%	14	0.872	0.761
BM1706	100%	100%	16	0.660	0.436
BM1225	100%	100%	20	0.433	0.187
BM4107	100%	100%	20	0.363	0.132
BM1905	98%	98%	23	0.495	0.245
BM47	98%	98%	23	0.729	0.531
ILSTS102	96%	96%	25	0.531	0.282
BMS510	96%	96%	28	0.466	0.217

The major allele frequency for each STR was determined based on an extensive library of 10–17,000 plains bison, and used to estimate the probability of identity (2.318 × 10^− 6^)

Regression analyses were performed to evaluate the relationship between the efficiency of each marker and several physical and chemical properties of the STR loci such as the location on the chromosome, length of the motif, C/G content, primer attributes and secondary structure (Additional file 4).

Heterozygosity (H_O_) was calculated both by animal and by locus. Heterozygosity estimates were calculated using fecal samples and blood samples separately, and the differences in heterozygosity were calculated based on samples that produced readable data for both. Overall statistical significance between all fecal and blood samples and pairwise statistical significance for each animal and marker was tested using a paired Student’s t-test.

## Results

**Fig. 1:**
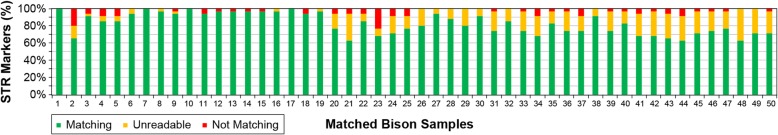
Efficiency of each pair of bison fecal and blood samples at 35 STR loci. The percentage of markers that matched for each pair, the percentage of unreadable results due to no amplification or unclear genotyping results, and the percentage of non-matching pairs where the blood and fecal samples from the same bison yielded different genotyping results are shown. Each fecal-blood pair from the same bison is referred to by number 1–50 (Additional file 1)

DNA from the 50 paired fecal and blood samples were analyzed at 35 STR loci. On average, 82.80% of the samples matched (range: 62.86–100%) at the markers tested, 13.31% yielded unreadable data (range: 0–37.14%), and the fecal and blood samples did not match at 3.89% of the markers (range: 0–22.86%) (Fig. 1).

**Fig. 2:**
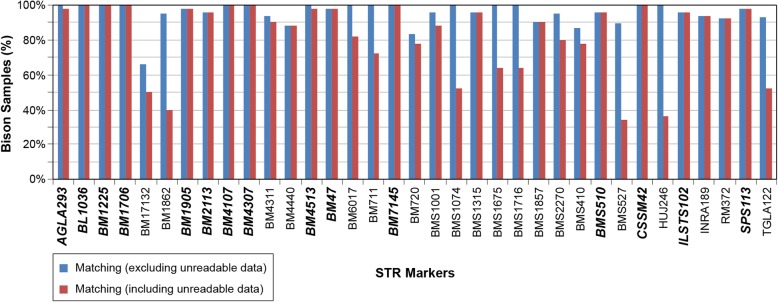
Efficiency of each STR marker for the 50 paired bison fecal and blood samples. The bars represent the percentage of samples that matched for each of the markers. The STR markers chosen for the final panel are shown in bold and italics

The percent of matching pairs for each STR marker was also determined by two methods (Fig. 2). The efficiency was calculated as the proportion of samples for each marker where the blood and fecal samples matched (Percent matching (including unreadable data)), as well as the proportion of the samples that produced readable data divided by the sum of those that either matched or did not match (Percent matching (excluding unreadable data)). The difference between the results from the two methods is the percent of unreadable samples.

The allelic dropout rate, calculated as the instances where only one of the two alleles in a blood sample were present in the matching fecal sample, was 0.023 overall, which means allelic dropout accounted for 60.3% of all mismatches between fecal and blood samples observed in this study. In order to assess potential contamination, cases were also examined where neither allele matched at a certain marker and the false allele rate was determined to be 0.0046, accounting for only 11.8% of all mismatches. The remainder of the mismatches were attributed to other types of discrepancies, such as when an individual appeared to be a heterozygote based on fecal genotypes, whereas the blood-based genotype was homozygous for one of the alleles. Therefore, in this example they still share an allele but the second allele from the fecal-derived DNA can be assumed to be an artifact.

Fifteen of the 35 STRs were chosen for a final panel of markers that performed at an exceptionally high efficiency with over 95% of the paired fecal and blood samples matching (Table 1). The 15 STR markers reside on 10 chromosomes, and the closest STRs on the same chromosome are located 14.76 Mb (18.15 cM) apart on chromosome 2. The probability of identity (P_ID_), after accounting for linkage, was calculated as 2.318 × 10^− 6^.

We have investigated a number of different parameters to identify the factors responsible for the discrepancy in the efficiency of different markers when tested on fecal material. However, no relationship was observed between the efficiency of the STR markers and primer length (R^2^ = 0.0016, *p* = 0.82), average primer G/C content (R^2^ = 0.0087, *p* = 0.60), amplicon length (R^2^ = 4 × 10^− 5^, *p* = 0.97), amplicon G/C content (R^2^ = 0.0051, *p* = 0.69), primer melting temperature (R^2^ = 0.018, *p* = 0.27), number of hairpin bases (R^2^ = 0.0154, *p* = 0.31), homodimers (R^2^ = 0.0041, p = 0.60), or heterodimers (R^2^ = 0.0052, *p* = 0.55) (Additional file 4). These calculations were based on the *Bos taurus* genome (UMD 3.1), and amplicon length and G/C content may differ slightly in bison. Because all bovid autosomal chromosomes are acrocentric, and the cattle reference assemblies were built starting from the centromere, we could assess if the distance of the STR regions from the centromere had any effect on the efficiency of the STR marker. However, no relationship was detected (R^2^ = 0.0465, *p* = 0.22) (Additional file 3).

**Fig. 3:**
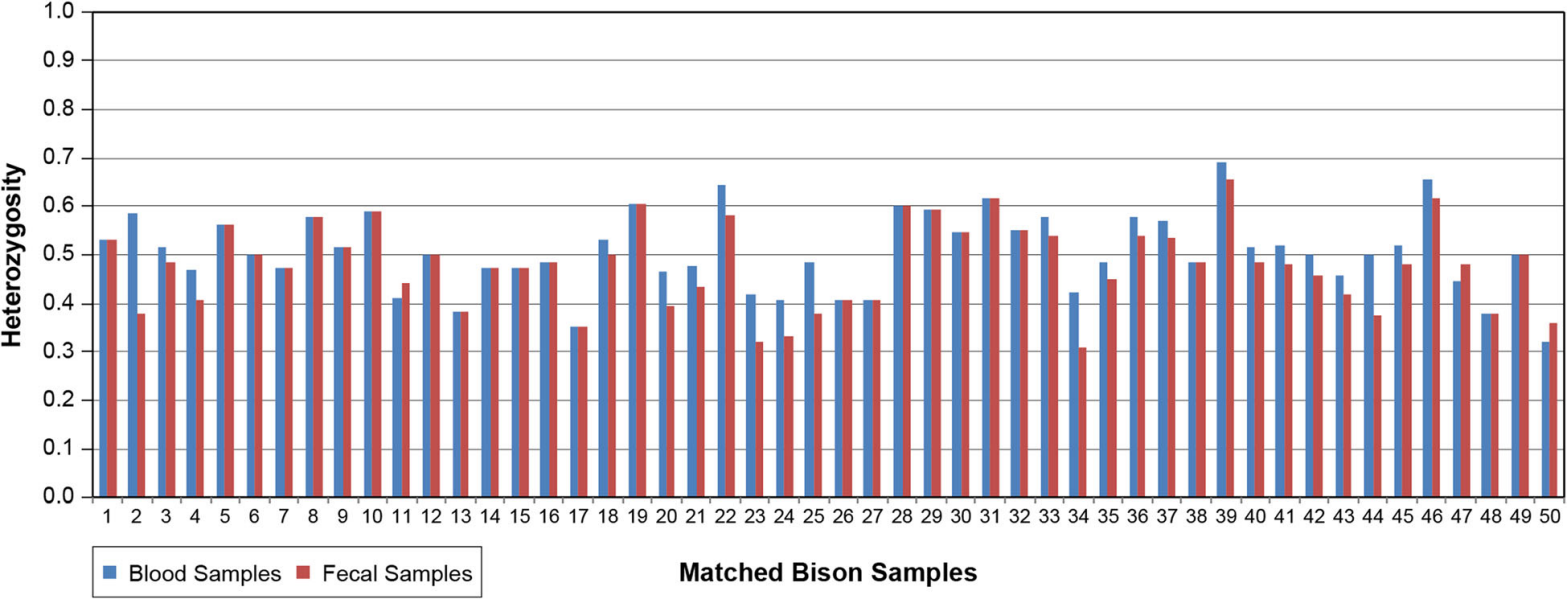
Heterozygosity estimates from blood and fecal samples by animal. A significant overall reduction in heterozygosity is seen when fecal samples are used (*p* < 0.0001). Each fecal-blood pair from the same bison is referred to by number 1–50 (Additional file 1)

**Fig. 4:**
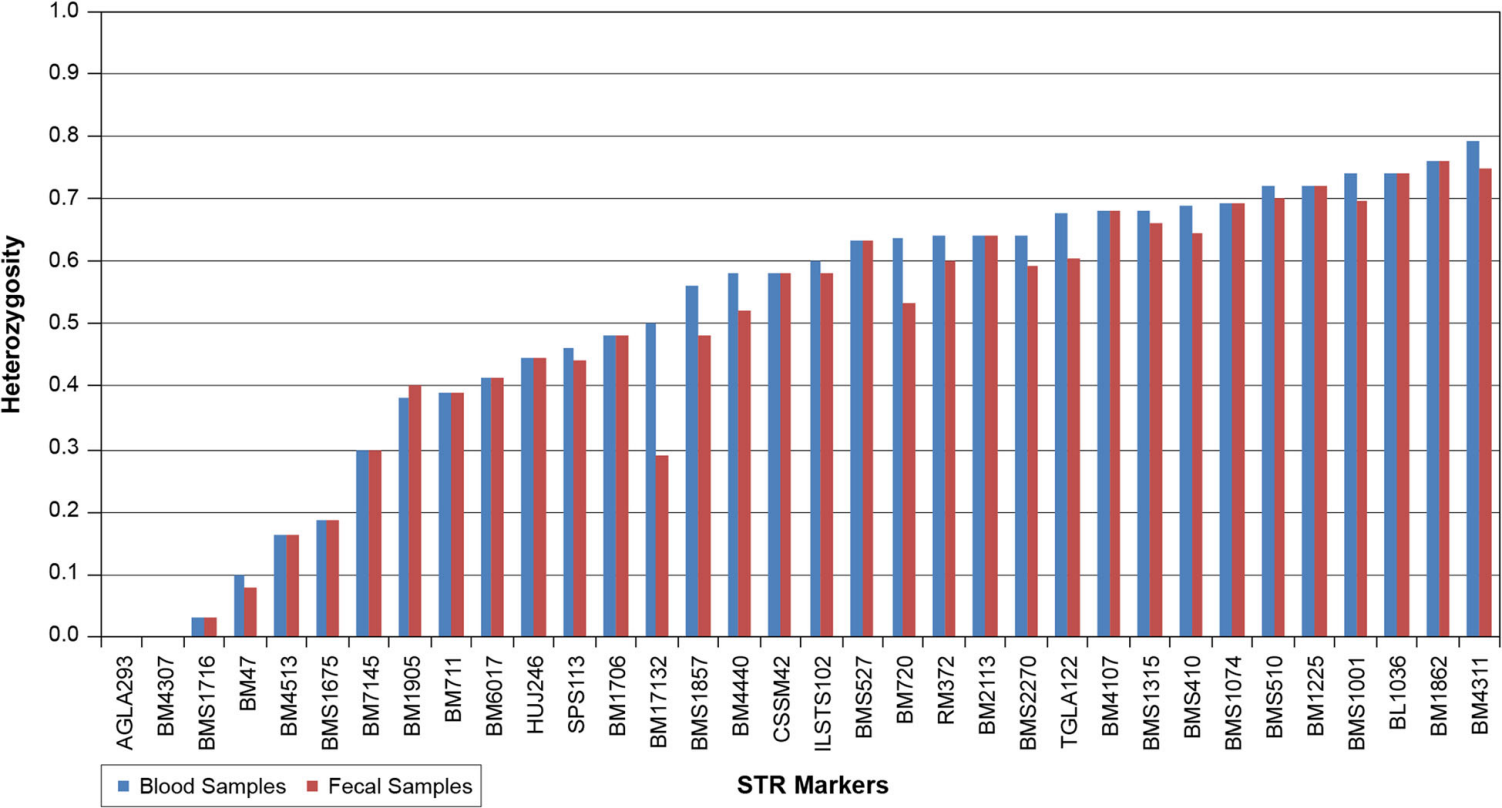
Heterozygosity estimates from blood and fecal samples by STR marker. A significant reduction is seen when fecal samples are used (*p* < 0.005)

Heterozygosity estimates were compared between fecal and blood samples and calculated in two ways: (i) what percent of loci were heterozygous in each bison (heterozygosity by animal, Fig. 3), and (ii) what percent of animals were heterozygous for each locus (heterozygosity by locus, Fig. 4). The hemizygous Y chromosome marker (INRA189) was excluded from this analysis.

For heterozygosity by animal, overall, the estimates from the fecal samples showed a significant 2.7% reduction in heterozygosity (*p* < 0.0001***) compared to the estimates from blood samples (Fig. 3). Nearly half of the animals showed no changes in heterozygosity, while others experienced a reduction of up to 20.7% (*p* < 0.05*). There were 3 samples where the heterozygosity estimates were actually higher in fecal samples, but none of those differences were significant (Pairs 11, 47, and 50).

Similarly, there was a significant overall reduction in fecal-derived heterozygosity by locus (*p* < 0.005**) (Fig. 4). Over half of the loci showed no change in heterozygosity between the blood and the fecal samples, while others showed a significant reduction as high as 21.1% (*p* < 0.05*). Only a single STR locus showed an increase in heterozygosity from fecal samples (BM1905) but it was not significant.

## Discussion

We evaluated 35 STR markers for use with fecal DNA by comparing genotypes generated from paired blood and fecal samples from 50 bison and investigated how heterozygosity is affected by using different types of samples. We concluded that the majority of mismatches between fecal and blood DNA were caused by allelic dropout, which has been previously identified as a major drawback to using fecal DNA [5, 21]. Yet, fecal marker validation beyond simply re-analyzing the poor quality fecal samples has remained rare.

During DNA extraction, bison hairs were found in many fecal samples. The origin of these hair samples is unknown; they could be from the same individual or from other bison. The source of these hairs could either be hair ripped out during sample collection or ingested during grazing or grooming. While extreme care was taken to avoid hairs during DNA extraction, some shed hair follicles may be a source of DNA during amplification. Cases where neither allele matched were rare (0.46% of cases) and explained only 12.12% of mismatched genotypes between blood and fecal paired samples. Due to the rarity of this event, and the fact that it was not a consistent mismatch across multiple loci in the same fecal-blood pairs, we can conclude that it is highly unlikely that foreign DNA has been amplified. This supports the notion that either the presence of hair follicle DNA did not affect our analysis, or that the hair in the fecal samples came from that individual.

A single Y chromosome marker was tested in the study (INRA189), however, we discourage using fecal DNA with any presence-absence markers where the absence of signal is a genotype in and of itself. A lack of signal could be misinterpreted as a null allele (in this case, a female bison), while it may have been the result of a failure in the amplification of the allele from fecal DNA. Thus, sex can only be determined with some level of certainty in cases where a heterozygous X chromosome marker and no amplification on the Y can be used to definitively identify some females, while Y chromosome amplification and only one X chromosome allele amplifying (hemizygosity) is strong evidence for a male. It is important to note that neither our X (BM6017) nor our Y chromosome (INRA189) markers performed with high enough reliability to be included in our final bison panel.

After careful comparison with paired blood samples, 15 of the 35 STRs were identified as highly reliable markers for bison genotyping using fecal material. However, verifying fecal markers by merely repeating the extraction or the amplification step can lead to erroneous results. Given that the number of bison in North America is currently around 500,000, these 15 markers yield a sufficiently low probability of identity for use in population genetics studies [42].

## Conclusions

We compared heterozygosity based on fecal- and blood-derived DNA and conclude that there is a highly significant (*p* < 0.005) decrease in heterozygosity estimates from fecal samples. Our results show that without a careful assessment of the markers, significantly skewed population metrics may be reported. A significant proportion of conservation genetics studies use fecal samples in animal species that are rare or endangered because in many of those cases, invasive sampling is not feasible [43–45]. Fecal DNA can lead to an artificially decreased heterozygosity estimate, which may negatively skew estimates of the genetic diversity of the species. This discrepancy in population metrics emphasizes the need for better quality control in the form of paired high-quality DNA samples to verify the reliability of each marker. We advise researchers to first test their markers by using a direct comparison between the low and high quality sources of DNA in a subset of their samples to validate those markers for use in low quality samples. Once verified, they can use those markers with a high level of confidence in the rest of their samples without the need to collect DNA from other sources.

In conclusion, careful consideration needs to be taken when designing fecal DNA studies. While numerous methods are used to evaluate genotyping error rates, we contend that a paired sample-based genotyping method such as the one described here will provide the most accurate validation of fecal DNA markers. This method will also yield the best sampling protocol and the most precise results. We present a set of 15 reliable STR markers for use with bison fecal DNA. Based on these verified markers, a population-wide study can be conducted using fecal DNA to evaluate genetic diversity parameters and population metrics in Yellowstone bison whose capture is unfeasible inside the national park. It would also aid with the evaluation of other herds that are under protection or lack the infrastructure to capture bison and acquire high quality DNA samples for analysis. While amplification was possible from DNA extracted from every fecal sample, some markers amplified more reliably than others. Due to the lack of consensus on what factors influence marker performance, the rigorous assessment of markers used in all fecal DNA studies is necessary to provide valid results. By comparing fecal samples to blood from the same animals we show a significant reduction (in some cases in excess of 20%) in heterozygosity, a common population genetics metric often used in fecal DNA studies. Our case study accentuates the need in fecal DNA studies concerning all animals, not just bison, to verify individual markers and fecal samples with a matching high quality DNA sample to validate each marker.

## Additional files

Additional file 1: List of the 50 pairs of fecal and blood samples from Yellowstone bison. (XLSX 11 kb)

Additional file 2: The list of the 35 STR markers used in the study. (XLSX 13 kb)

Additional file 3: Statistical analysis and raw genotyping data. (XLSX 87 kb)

Additional file 4: Efficiency of STR markers based on various parameters tested. (JPG 137 kb)

## Abbreviations

cM: Centimorgan
DNA: Deoxyribonucleic acid
dNTP: Deoxynucleotide
LOD: Likelihood of odds
Mb: Megabase
PCR: Polymerase chain reaction
P_ID_: Probability of identity
STR: Short tandem repeat
