# Supplementary figures and images for: Evaluation of fecal samples as a valid source of DNA by comparing paired blood and fecal samples from American bison (Bison bison)
Source: BMC Genet. 2019 Feb 26;20:22. doi: 10.1186/s12863-019-0722-3 (PMC6390568; doi:10.1186/s12863-019-0722-3)

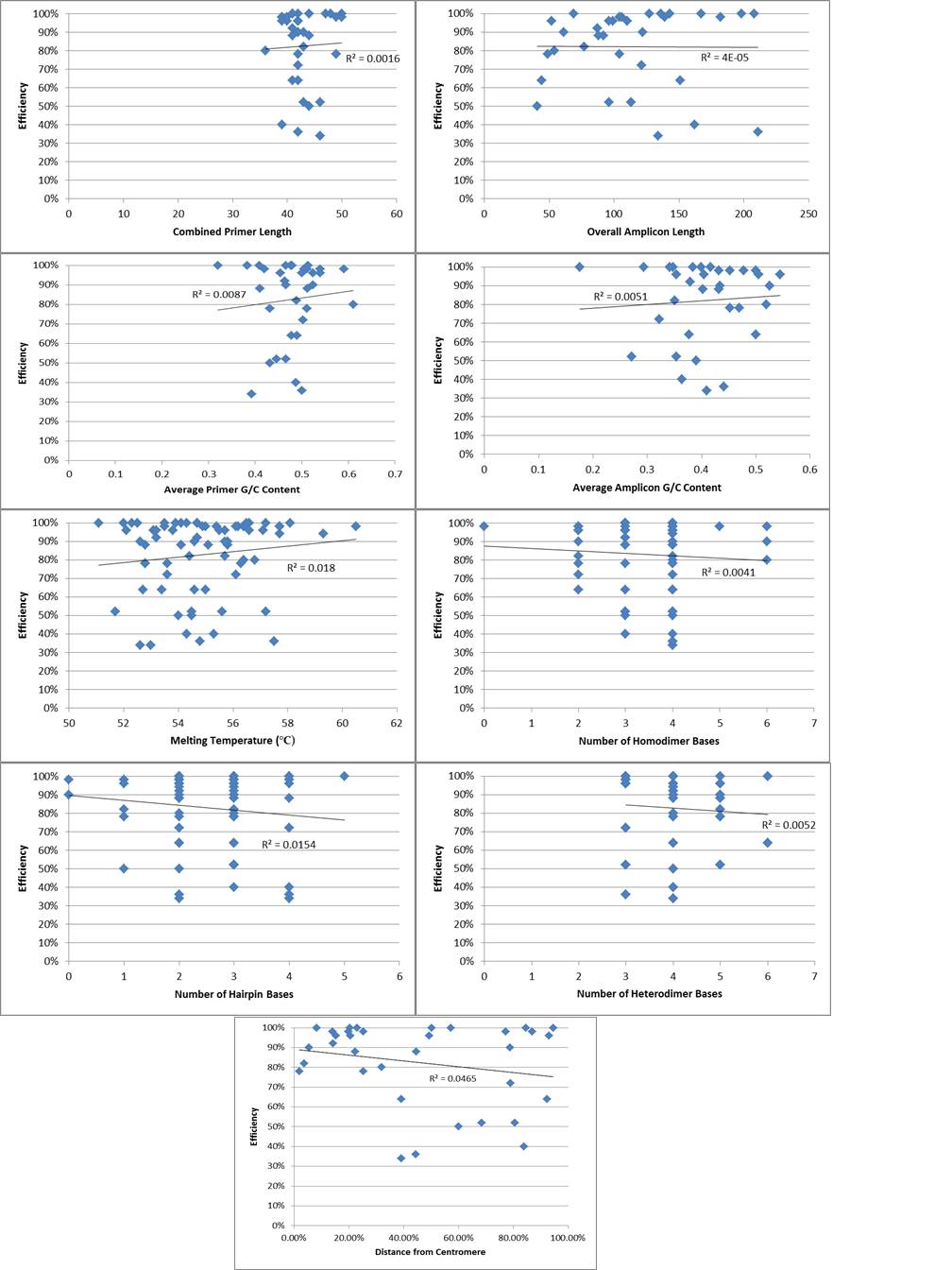

Supplement: Supplementary file 4 — Efficiency of STR markers based on various parameters tested. (JPG 137 kb) [file 12863_2019_722_MOESM4_ESM.jpg]
